# Dynamical Behaviors of a Translating Liquid Crystal Elastomer Fiber in a Linear Temperature Field

**DOI:** 10.3390/polym14153185

**Published:** 2022-08-04

**Authors:** Lin Zhou, Wangyang Yu, Kai Li

**Affiliations:** 1 School of Mechanical and Electrical Engineering, Anhui Jianzhu University, Hefei 230601, China; 2 School of Civil Engineering, Anhui Jianzhu University, Hefei 230601, China

**Keywords:** liquid crystal elastomer, fiber, translating, heat-driven, dynamics, constitutive model

## Abstract

Liquid crystal elastomer (LCE) fiber with a fixed end in an inhomogeneous temperature field is capable of self-oscillating because of coupling between heat transfer and deformation, and the dynamics of a translating LCE fiber in an inhomogeneous temperature field are worth investigating to widen its applications. In this paper, we propose a theoretic constitutive model and the asymptotic relationship of a LCE fiber translating in a linear temperature field and investigate the dynamical behaviors of a corresponding fiber-mass system. In the three cases of the frame at rest, uniform, and accelerating translation, the fiber-mass system can still self-oscillate, which is determined by the combination of the heat-transfer characteristic time, the temperature gradient, and the thermal expansion coefficient. The self-oscillation is maintained by the energy input from the ambient linear temperature field to compensate for damping dissipation. Meanwhile, the amplitude and frequency of the self-oscillation are not affected by the translating frame for the three cases. Compared with the cases of the frame at rest, the translating frame can change the equilibrium position of the self-oscillation. The results are expected to provide some useful recommendations for the design and motion control in the fields of micro-robots, energy harvesters, and clinical surgical scenarios.

## 1. Introduction

The phenomenon of continuous periodic motion of a system under the influence of a steady external environment stimulation is known as self-excited motion [1,2,3,4]. To compensate for the energy dissipation consumed by the damping, the periodic motion of self-excited oscillation is maintained by collecting the energy directly from the external environment [5,6,7]. This property minimizes the complexity of self-excited oscillation systems, making complicated control system design easier to implement and allowing for high load capacity [8,9]. Self-excited motion possesses the autonomous characteristics [10,11,12,13,14], which aids in the study of non-equilibrium thermodynamic processes [15,16], and has several applications in the fields of active machinery [17,18,19,20], mobile robots [21,22], energy acquisition [23,24,25,26], and motors [27].

Self-oscillation phenomena have been reported based on several active materials, such as liquid crystal elastomer (LCE) [28,29,30,31], polyelectrolyte gel [32,33], and hydrogel [34,35]. When subjected to external excitations such as light [6], chemicals [34], electric field [36], magnetic field [37], and heat [38], these responsive materials can change their own shape and locomote. Based on various kinds of stimuli-responsive materials, a large number of modes of self-excited motion have also been constructed, such as rolling [12,18,20,39], bending [40,41,42,43], vibration [44,45], stretching and shrinking [46,47], torsion [7,48], swinging [49,50], swimming [51], buckling [29,52,53,54], jumping [45,55,56], rotation [57], eversion or inversion [38,58], and even self-excited synchronized motion of some coupled liquid crystalline oscillators [59]. The mechanisms of these self-excited motions are explained by the nonlinear feedback mechanisms of the systems, such as the self-shadowing mechanism [18,60], the coupling mechanism among liquid volatilization and membrane deformation [16], and a combination of finite deformation and chemical reaction [32,33].

One of the prevalent external stimuli for triggering the actuation of responsive materials is heat [61,62,63]. In a steady-temperature field, self-excited oscillation of a liquid crystal elastomer fiber with a hanging weight was recently reported [46]. In the experiment, the anisotropic rod-like liquid crystalline molecules and stretchy long-chain polymers are used to synthesize and prepare the LCE material. The LCE microfiber is hung from a rigid flat plate in the temperature field and the mass is placed on the bottom free end of the fiber. The fiber system was discovered to be able to vibrate continuously in an inhomogeneous temperature field. Theoretical research demonstrates that the self-oscillation is caused by the combination between the vibration of the fiber and heat exchange [47]. When the fiber vibrates, it can collect heat energy from the environment, to compensate for the damping dissipation and keep its own motion [47]. This complex, thermodynamic-coupled nonlinear dynamics problem is further studied in [61]. It abstractly presented a fiber engine module and established its constitutive model and asymptotic relationship, which is similar to the Kelvin-Voigt viscoelastic model consisting of a dashpot and a spring. It is found that the one-end-fixed fiber in a linear temperature field has three kinds of behaviors: damper, spring, and engine.

Although considerable research has been carried out on the self-oscillation motion of a thermal-responsive LCE fiber with one end fixed [46,47,61], the dynamics of a translating thermally responsive fiber with a moving frame need to be explored for further possible applications. To investigate the dynamical behaviors of the LCE fiber translating in a temperature field varying linearly with position, we propose a theoretic constitutive model of LCE fiber and its asymptotic relationship and study the fiber-mass system in three typical cases of the frame, at rest, uniform, and accelerating translation. The objective of this work is to establish a constitutive model, obtain its dynamical behavior, and provide some useful recommendations for the design and motion control of micro-robots of various structures and applications.

The article is organized as follows. In Section 2, a constitutive model of translating LCE fiber in a linear temperature field is proposed, and its asymptotic relationship is derived for the small characteristic time. In Section 3, we establish a thermally responsive fiber-mass system with zero characteristic time, and formulate its corresponding governing equations based on the constitutive model, to investigate its dynamical behaviors for three typical cases of the frame, at rest, uniform, and accelerating translation. In Section 4, by considering the small characteristic time, we study the dynamics of the fiber-mass system through the proposed constitutive model and asymptotic relationship. In Section 5, we further investigate the dynamics of the fiber-mass system with a finite characteristic time based on the constitutive model. Finally, a short summary is provided in Section 6.

## 2. Constitutive Model of a Translating Thermally Responsive Fiber

In this section, we propose a constitutive model of a LCE fiber connected with a translating frame in a linear temperature field. For a small characteristic time, its asymptotic relationship is further derived. When the displacements and velocities of the two ends of the LCE fiber are given, the tensional force in the fiber can be calculated. 

### 2.1. Constitutive Model

**Figure 1:**
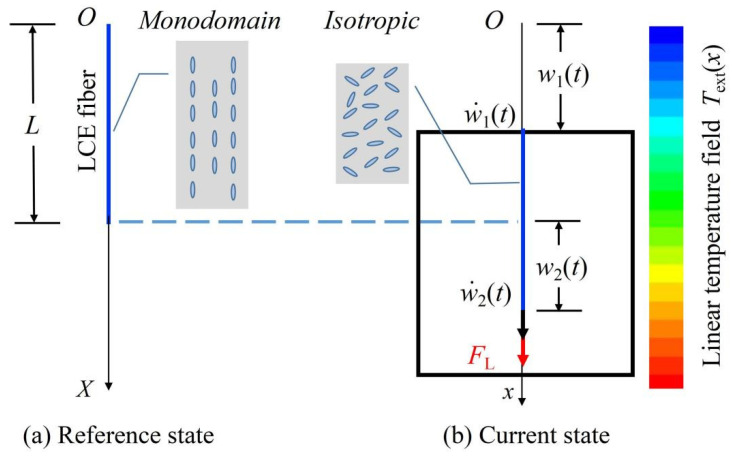
Schematic model for a LCE fiber connected with a translating frame in a linear temperature field. (**a**) Reference state; (**b**) Current state. The original length of the fiber is L. $w_{1}\left(t\right)$, ${\dot{w}}_{1}\left(t\right)$, $w_{2}\left(t\right)$, and ${\dot{w}}_{2}\left(t\right)$ are the displacements and velocities of the translating frame and the free end of the LCE fiber. When the displacements and velocities of the two ends of the LCE fiber are given, the tension force of the fiber can be determined.

The model for a LCE fiber connected with a translating frame in a linear temperature field is shown in Figure 1. We define a reference state of the fiber, which represents the free-standing fiber without thermal expansion at reference temperature $T_{r}$, and the original length is denoted by L. $w_{1}\left(t\right)$, ${\dot{w}}_{1}\left(t\right)$, $w_{2}\left(t\right)$, and ${\dot{w}}_{2}\left(t\right)$ are the displacements and velocities of the translating frame and the free end of the LCE fiber, which is regarded as a current state. To analyze the deformation of a translating LCE fiber, a Lagrangian coordinate system, X, is fixed in the reference configuration of the fiber, and the Eulerian coordinate system, x, is also built in the current state to describe the spatial coordinate. The origins of coordinates are fixed at the O point. Meanwhile, $x=x\left(X,t\right)$ is the instantaneous position of a material at point X of the translating fiber, and $u\left(X,t\right)$ is its instantaneous displacement. 

LCE material can generally generate a rapid deformation response and recoverable deformation, which is synthesized and prepared by liquid crystalline molecules and flexible long-chain polymers [46]. When a tensile force is applied onto the LCE fiber, it is stretched in the monodomain state. When heated up in the temperature field, the stretched fiber contracts along the axial direction because of the transition from the monodomain phase to the isotropic phase. When cooled down, the fiber can completely relax to its original length. Hence, the nematic–isotropic phase transition and the reorientation of the liquid crystal mesogens occur during the contraction and expansion of the fiber. For simplicity, we assume that the tension force, $F_{L}\left(t\right)$, of the translating fiber is proportional to its elastic strain, as:

(1) $$F_{L}\left(t\right)=KL [\varepsilon \left(X,t\right)-\varepsilon_{T}\left(X,t\right)],$$

in which K is the spring constant. $\varepsilon \left(X,t\right)$ is the one-dimensional strain, namely,

(2) $$\varepsilon \left(X,t\right)=\frac{\partial u\left(X,t\right)}{\partial X}.$$

$\varepsilon_{T}\left(X,t\right)$ is the thermally induced strain, and we also assume that it is proportional to the temperature difference, $T\left(X,t\right)$, in the fiber between the actual temperature of the fiber and the reference temperature $T_{r}$, i.e.,

(3) $$\varepsilon_{T}\left(X,t\right)=\alpha T\left(X,t\right),$$

with α being the thermal expansion coefficient. α<0 indicates thermal contraction, while α>0 indicates thermal expansion.

Considering that in the fiber, $F_{L}\left(t\right)$ is uniform and constant, Equation (1) is integrated on both sides from 0 to X. By combining Equations (2) and (3), Equation (1) can further be expressed as:

(4) $$F_{L}\left(t\right)X=KL [u\left(X,t\right)-w_{1}\left(t\right)-\alpha \int_{0}^{X}T\left(X,t\right)dX].$$

When X=L, the tensional force at the end of the translating fiber can be reduced as:

(5) $$F_{L}\left(t\right)=K [w_{2}\left(t\right)-w_{1}\left(t\right)-\alpha \int_{0}^{L}T\left(X,t\right)dX].$$

It is noted that the temperature field in the translating fiber varies with time and is inhomogeneous. Hence, there exits heat exchange between the translating fiber and the surrounding environment, and we denote the temperature distribution by $T_{\mathrm{ext}}\left(x\right)$. We also assume that the thin fiber’s radius, R, is significantly smaller than its length, L. As a result, the temperature field in the translating fiber is essentially homogeneous over its radius. The temperature field, $T=T\left(X,t\right)$, in the fiber is governed by:

(6) $$\tau \frac{dT\left(X,t\right)}{dt}=T_{\mathrm{ext}}\left(x\right)-T\left(X,t\right),$$

where τ is the characteristic time, which is related to the heat capacity of the fiber per unit length, $\rho_{c}$, and the heat exchange coefficient, h. The characteristic time can be expressed by $\tau =\rho_{c}/h$. The actual external temperature field is generally complex and nonlinear and may be produced by a light on–off or hot plate. In this study, for simplicity, we assume that the external steady temperature field is linear with the spatial coordinate, and the distribution of the linear temperature field can be described by:

(7) $$T_{\mathrm{ext}}\left(x\right)=\beta x,$$

where β is the temperature gradient. It is noted that the origin of spatial coordinates is fixed at O point (x=0), and the temperature difference, $T_{\mathrm{ext}}\left(0\right)$, is zero, i.e., the actual external temperature is equal to the reference temperature $T_{r}$. It should be noted that the spatial coordinate of the material point, $x\left(X,t\right)$, can be calculated by:

(8) $$x\left(X,t\right)=u\left(X,t\right)+X,$$

where $u\left(X,t\right)$ can be derived from Equations (4) and (5) as:

(9) $$u\left(X,t\right)=\frac{X}{L} [w_{2}\left(t\right)-w_{1}\left(t\right)-\alpha \int_{0}^{L}T\left(X,t\right)dX]+\alpha \int_{0}^{X}T\left(X,t\right)dX+w_{1}\left(t\right).$$

By introducing the following dimensionless parameters: $\bar{t}=t/\sqrt{L/g}$, ${\bar{F}}_{L}=F_{L}/mg$, $\bar{u}=u/L$, ${\bar{w}}_{1}=w_{1}/L$, ${\bar{w}}_{2}=w_{2}/L$, $\bar{X}=X/L$, $\bar{x}=x/L$, $\bar{\tau }=\tau /\sqrt{L/g}$, $\bar{K}=KL/mg$, $\bar{\alpha }=\alpha T_{L}$, $\bar{T}=T/T_{L}$, ${\bar{T}}_{\mathrm{ext}}=T_{\mathrm{ext}}/T_{L}$, and $\bar{\beta }=\beta L/T_{L}$ ($T_{L}$ is the environmental temperature at x=L), we combine Equations (5)–(9) and derive the formulas as follows:

(10) $$\bar{\tau }\frac{d\bar{T}\left(\bar{X},\bar{t}\right)}{d\bar{t}}=\bar{\beta }\left\{\bar{X} [{\bar{w}}_{2}\left(\bar{t}\right)-{\bar{w}}_{1}\left(\bar{t}\right)-\bar{\alpha }\int_{0}^{1}\bar{T}\left(\bar{X},\bar{t}\right)d\bar{X}]+\bar{\alpha }\int_{0}^{\bar{X}}\bar{T}\left(\bar{X},\bar{t}\right)d\bar{X}+{\bar{w}}_{1}\left(\bar{t}\right)+\bar{X}\right\}-\bar{T}\left(\bar{X},\bar{t}\right),$$

(11) $${\bar{F}}_{L}\left(\bar{t}\right)=\bar{K} [{\bar{w}}_{2}\left(\bar{t}\right)-{\bar{w}}_{1}\left(\bar{t}\right)-\bar{\alpha }\int_{0}^{1}\bar{T}\left(\bar{X},\bar{t}\right)d\bar{X}].$$

Equation (10) shows that the temperature field inside the fiber can be determined for a given displacement of the two ends of the fiber. Then, the tension force of the fiber can be calculated using Equation (11). In this study, the constitutive model of the translating fiber is considered Equations (10) and (11). The four dimensionless parameters, such as the spring constant $\bar{K}$, the temperature gradient $\bar{\beta }$, the characteristic time $\bar{\tau }$, and the thermal expansion coefficient $\bar{\alpha }$, are important influencing factors of the constitutive model. Conveniently, the constitutive model can be used to analyze the translating fiber-mass system in the following sections.

### 2.2. Asymptotic Relationship 

In the following, we develop the asymptotic relationship of the translating LCE fiber with a small characteristic time, $\bar{\tau }$, which means the situations of a high heat transfer coefficient or a low heat capacity. For $\bar{\tau }<<1$, the temperature field can be written as:

(12) $$\bar{T}\left(\bar{X},\bar{t}\right)={\bar{T}}^{\left(0\right)}\left(\bar{X},\bar{t}\right)+\bar{\tau }{\bar{T}}^{\left(1\right)}\left(\bar{X},\bar{t}\right)+O\left({\bar{\tau }}^{2}\right).$$

Then, we insert Equation (12) into Equation (10) and compare the coefficients of the same order of $\bar{\tau }$. The temperature field can be further given as:

(13) $$\bar{\beta }\bar{X}{\bar{w}}_{2}\left(\bar{t}\right)-\bar{\alpha }\bar{\beta }\bar{X}\int_{0}^{1}{\bar{T}}^{\left(0\right)}\left(\bar{X},\bar{t}\right)d\bar{X}+\bar{\alpha }\bar{\beta }\int_{0}^{\bar{X}}{\bar{T}}^{\left(0\right)}\left(\bar{X},\bar{t}\right)d\bar{X}-\bar{\beta }\bar{X}{\bar{w}}_{1}\left(\bar{t}\right)+\bar{\beta }{\bar{w}}_{1}\left(\bar{t}\right)+\bar{\beta }\bar{X}-{\bar{T}}^{\left(0\right)}\left(\bar{X},\bar{t}\right)=0,$$

(14) $$\frac{d{\bar{T}}^{\left(0\right)}\left(\bar{X},\bar{t}\right)}{d\bar{t}}=-\bar{\alpha }\bar{\beta }\bar{X}\int_{0}^{1}{\bar{T}}^{\left(1\right)}\left(\bar{X},\bar{t}\right)d\bar{X}+\bar{\alpha }\bar{\beta }\int_{0}^{\bar{X}}{\bar{T}}^{\left(1\right)}\left(\bar{X},\bar{t}\right)d\bar{X}-{\bar{T}}^{\left(1\right)}\left(\bar{X},\bar{t}\right).$$

By analytically solving Equation (13), we can obtain the zero-order part of the temperature field as follows:

(15) $${\bar{T}}^{\left(0\right)}\left(\bar{X},\bar{t}\right)=\frac{\bar{\beta } [{\bar{w}}_{2}\left(\bar{t}\right)-{\bar{w}}_{1}\left(\bar{t}\right)+1]}{e^{\bar{\alpha }\bar{\beta }}-1}\left(e^{\bar{\alpha }\bar{\beta }X}-1\right)+\bar{\beta }{\bar{w}}_{1}\left(\bar{t}\right).$$

Then, we combine Equation (14) with Equation (15), and the first-order part of the temperature field can be calculated as:

(16) $${\bar{T}}^{\left(1\right)}\left(\bar{X},\bar{t}\right)=\frac{\bar{\beta }\left({\bar{\dot{w}}}_{2}\left(\bar{t}\right)-{\bar{\dot{w}}}_{1}\left(\bar{t}\right)\right)}{e^{\bar{\alpha }\bar{\beta }}-1} [\frac{\left(e^{\bar{\alpha }\bar{\beta }\bar{X}}-1\right)\left(1-e^{\bar{\alpha }\bar{\beta }}+\bar{\alpha }\bar{\beta }e^{\bar{\alpha }\bar{\beta }}\right)}{e^{\bar{\alpha }\bar{\beta }}-1}-\bar{\alpha }\bar{\beta }\bar{X}e^{\bar{\alpha }\bar{\beta }\bar{X}}].$$

Therefore, the temperature field can be solved as:

(17) $$\begin{array}{l} \bar{T}\left(\bar{X},\bar{t}\right)=\frac{\bar{\beta } [{\bar{w}}_{2}\left(\bar{t}\right)-{\bar{w}}_{1}\left(\bar{t}\right)+1]}{e^{\bar{\alpha }\bar{\beta }}-1}\left(e^{\bar{\alpha }\bar{\beta }X}-1\right)+\bar{\beta }{\bar{w}}_{1}\left(\bar{t}\right)\\ +\bar{\tau }\frac{\bar{\beta }\left({\bar{\dot{w}}}_{2}\left(\bar{t}\right)-{\bar{\dot{w}}}_{1}\left(\bar{t}\right)\right)}{e^{\bar{\alpha }\bar{\beta }}-1} [\frac{\left(e^{\bar{\alpha }\bar{\beta }\bar{X}}-1\right)\left(1-e^{\bar{\alpha }\bar{\beta }}+\bar{\alpha }\bar{\beta }e^{\bar{\alpha }\bar{\beta }}\right)}{e^{\bar{\alpha }\bar{\beta }}-1}-\bar{\alpha }\bar{\beta }\bar{X}e^{\bar{\alpha }\bar{\beta }\bar{X}}] \end{array}$$

We combine Equation (11) with Equation (17) and obtain the tensional force, $F_{L}\left(t\right)$, of the translating fiber as:

(18) $${\bar{F}}_{L}\left(\bar{t}\right)=\bar{K}\bar{\alpha }\bar{\beta }\bar{\tau }\frac{1-e^{\bar{\alpha }\bar{\beta }}+\bar{\alpha }\bar{\beta }e^{\bar{\alpha }\bar{\beta }}}{{\left(e^{\bar{\alpha }\bar{\beta }}-1\right)}^{2}} [{\bar{\dot{w}}}_{2}\left(\bar{t}\right)-{\bar{\dot{w}}}_{1}\left(\bar{t}\right)]+\frac{\bar{K}\bar{\alpha }\bar{\beta }}{e^{\bar{\alpha }\bar{\beta }}-1} [{\bar{w}}_{2}\left(\bar{t}\right)-e^{\bar{\alpha }\bar{\beta }}{\bar{w}}_{1}\left(\bar{t}\right)]+\bar{K}\left(\frac{\bar{\alpha }\bar{\beta }}{e^{\bar{\alpha }\bar{\beta }}-1}-1\right).$$

Equation (18) is the asymptotic relationship of the constitutive model of translating LCE fiber described by Equations (10) and (11). Next, we further establish a dynamic fiber-mass system model and utilize the constitutive model to obtain the corresponding governing equation. For three typical cases of the frame at rest, uniform, and accelerating translation, the asymptotic relationship is utilized in Section 3 to study the dynamical behaviors of the fiber-mass system with zero characteristic time, and both the constitutive model and approximate solution are used in Section 4 to study the case of a small characteristic time. The constitutive model is also utilized to investigate the case of finite characteristic time, τ, in Section 5. The typical values of material properties and geometric parameters from accessible experiments are listed in Table 1 [46,47], and the dimensionless parameters are estimated in Table 2.

## 3. Dynamics of the Fiber-Mass System with Zero Characteristic Time

In this section, we establish a thermally responsive fiber-mass system with zero characteristic time, and formulate its corresponding governing equations based on the constitutive model, to investigate its dynamical behaviors for three typical cases of the frame at rest, uniform, and accelerating translation. By obtaining the dynamical behavior of this translating thermally responsive fiber-mass system, we can provide some useful recommendations for the design and motion control of micro-robots of various structures and applications.

### 3.1. Governing Equation

**Figure 2:**
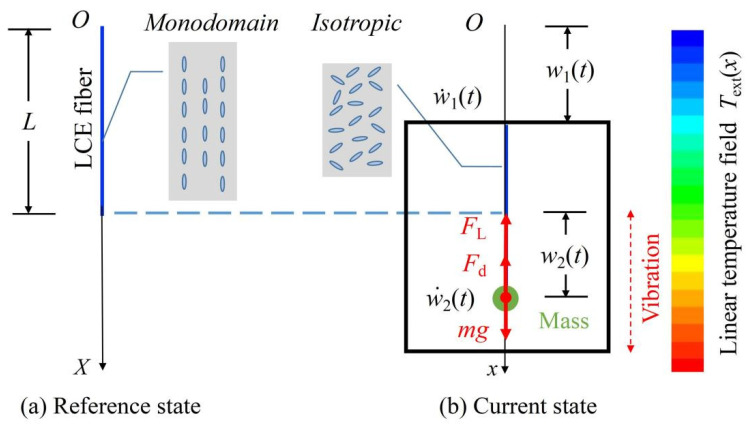
Schematic of the fiber-mass system translating in a linear temperature field. (**a**) Reference state; (**b**) Current state. A mass block with a mass of m is attached to the free end of the LCE fiber connected to a frame that ignores gravity. The motion of the fiber-mass system is determined by the displacement, velocity, and acceleration of the mass connected to the end of the LCE fiber, as well as the displacement of the frame attached to the other end of the LCE fiber.

We first study the dynamics of the fiber-mass system translating in a temperature field varying linearly with position for zero characteristic time, $\bar{\tau }=0$, which is sketched in Figure 2. One end of the LCE fiber is attached to a mass block with a mass of m, and the other end is connected to a frame that ignores gravity. Therefore, the governing equation for the dynamical model of the mass block can be built as below:

(19) $$m{\ddot{w}}_{2}\left(t\right)-mg+F_{L}\left(t\right)+F_{d}\left({\dot{w}}_{2}\right)=0,$$

where $F_{d}\left({\dot{w}}_{2}\right)$ is the damping force of the mass block due to the damping, mg is the gravitational force of the mass block, and $F_{L}\left(t\right)$ is the tensional force from the fiber. We assume that the damping force is linearly proportional to its velocity, which can be written as:

(20) $$F_{d}\left({\dot{w}}_{2}\right)=a_{0}{\dot{w}}_{2}.$$

where $a_{0}$ is the damping coefficient. By defining ${\bar{F}}_{d}=F_{d}/mg$ and ${\bar{a}}_{0}=\frac{a_{0}}{m}\sqrt{\frac{L}{g}}$, and submitting Equations (18) and (20) into Equation (19), the dimensionless form of the governing equation can be obtained as:

(21) $${\bar{\ddot{w}}}_{2}\left(t\right)+{\bar{a}}_{0}{\bar{\dot{w}}}_{2}\left(\bar{t}\right)+\frac{\bar{K}\bar{\alpha }\bar{\beta }}{e^{\bar{\alpha }\bar{\beta }}-1}{\bar{w}}_{2}\left(\bar{t}\right)-\frac{\bar{K}\bar{\alpha }\bar{\beta }e^{\bar{\alpha }\bar{\beta }}}{e^{\bar{\alpha }\bar{\beta }}-1}{\bar{w}}_{1}\left(\bar{t}\right)+\bar{K}\left(\frac{\bar{\alpha }\bar{\beta }}{e^{\bar{\alpha }\bar{\beta }}-1}-1\right)-1=0.$$

By defining ${\hat{w}}_{2}\left(\bar{t}\right)={\bar{w}}_{2}\left(\bar{t}\right)+\frac{e^{\bar{\alpha }\bar{\beta }}-1}{\bar{K}\bar{\alpha }\bar{\beta }} [\bar{K}\left(\frac{\bar{\alpha }\bar{\beta }}{e^{\bar{\alpha }\bar{\beta }}-1}-1\right)-1]$, the governing equation can be simplified as:

(22) $${\hat{\ddot{w}}}_{2}\left(\bar{t}\right)+{\bar{a}}_{0}{\hat{\dot{w}}}_{2}\left(\bar{t}\right)+\frac{\bar{K}\bar{\alpha }\bar{\beta }}{e^{\bar{\alpha }\bar{\beta }}-1}{\hat{w}}_{2}\left(\bar{t}\right)=\frac{\bar{K}\bar{\alpha }\bar{\beta }e^{\bar{\alpha }\bar{\beta }}}{e^{\bar{\alpha }\bar{\beta }}-1}{\bar{w}}_{1}\left(\bar{t}\right).$$

It can be seen from Equation (22) that the motion of the fiber-mass system is determined by the displacement, velocity, and acceleration of the mass connected to the end of the LCE fiber, as well as the displacement of the frame attached to the other end of the LCE fiber. With the help of Matlab software, we adopt the four-order Runge–Kutta method described in detail in [47,61] to solve the differential Equation (22) with variable coefficients. In this paper, we investigate the vibration behavior of the mass block for three typical cases of the frame at rest, uniform, and accelerating translation.

### 3.2. Static Frame with ${\bar{\dot{w}}}_{1}\left(\bar{t}\right)=0$

In the case of a static frame with ${\bar{\dot{w}}}_{1}\left(\bar{t}\right)=0$, Equation (22) can be simplified as:

(23) $${\hat{\ddot{w}}}_{2}\left(\bar{t}\right)+{\bar{a}}_{0}{\hat{\dot{w}}}_{2}\left(\bar{t}\right)+\frac{\bar{K}\bar{\alpha }\bar{\beta }}{e^{\bar{\alpha }\bar{\beta }}-1}{\hat{w}}_{2}\left(\bar{t}\right)=0.$$

Equation (23) is the standard form of the damped free vibration differential equation. By solving the equation, the solution can be analytically derived as:

(24) $${\hat{w}}_{2}\left(\bar{t}\right)=Ae^{-\delta \bar{t}}\sin\left(\sqrt{\omega_{0}^{2}-\delta^{2}}\bar{t}+\theta \right),$$

where the natural angular frequency $\omega_{0}=\sqrt{\frac{\bar{K}\bar{\alpha }\bar{\beta }}{e^{\bar{\alpha }\bar{\beta }}-1}}$, $\delta =\frac{{\bar{a}}_{0}}{2}$, and the parameters $A=\sqrt{x_{0}^{2}+\frac{{\left(v_{0}+\delta x_{0}\right)}^{2}}{\omega_{0}^{2}-\delta^{2}}}$ and $\theta =\arctan\left(\frac{x_{0}\sqrt{\omega_{0}^{2}-\delta^{2}}}{v_{0}+\delta x_{0}}\right)$ are related to the system’s initial condition setting, especially, ${\hat{w}}_{2}\left(0\right)=x_{0}$ and ${\hat{\dot{w}}}_{2}\left(0\right)=v_{0}$.

**Figure 3:**
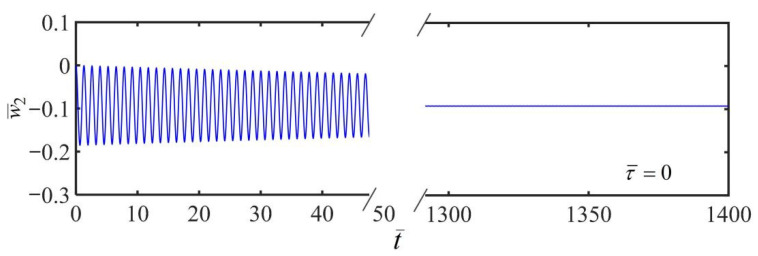
Variation of the displacement of the mass block with time during the damped free vibration of the system in a linear temperature field with a static frame. The vibration of the mass block stops in a static condition due to the damping consuming the energy.

The variation of the displacement of the mass with time for the initial condition of the system setting as ${\bar{w}}_{2}\left(0\right)=0$ and ${\bar{\dot{w}}}_{2}\left(0\right)=0$ is plotted in Figure 3 by using Equation (24). We set the system variables $\bar{K}=20$, $\bar{\alpha }=-0.3$, $\bar{\beta }=1$, and ${\bar{a}}_{0}=0.01$ for the numerical calculations. It is demonstrated that the mass block vibrates up and down when subjected to its own gravity and the tensional force of the fiber. Due to the damping consuming the energy, the amplitude of the vibration reduces dramatically over time and rapidly decays to zero.

### 3.3. Uniform Translational Frame with ${\bar{\dot{w}}}_{1}\left(\bar{t}\right)=C$

**Figure 4:**
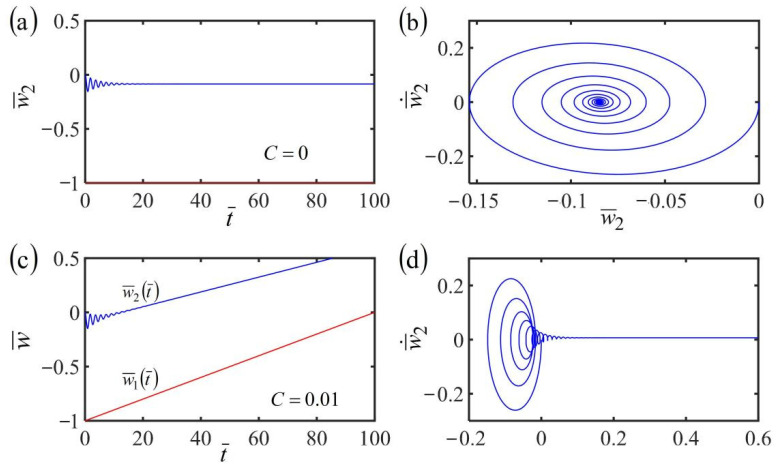
(**a**) Time history and (**b**) phase trajectory of the displacement of the system consisting of fiber and mass in the case of the frame at rest in a linear temperature field. (**c**) Time history and (**d**) phase trajectory of the displacement of the system consisting of fiber and mass in the case of the frame at uniform translation in a linear steady temperature field. The red line represents ${\bar{w}}_{1}\left(\bar{t}\right)$ and the blue line represents ${\bar{w}}_{2}\left(\bar{t}\right)$. The equilibrium position of mass and the length of the LCE fiber depend on the translation of the frame.

We further investigate the dynamics of a system consisting of a fiber and a mass in the case of a uniform translational frame with ${\bar{\dot{w}}}_{1}\left(\bar{t}\right)=C$. By setting the dimensionless parameters $\bar{K}=10$, $\bar{\alpha }=-0.38$, $\bar{\beta }=1$, and ${\bar{a}}_{0}=0.45$, Figure 4 plots the time histories of the displacements and phase trajectories in the cases of ${\bar{\dot{w}}}_{1}\left(\bar{t}\right)=0$ and ${\bar{\dot{w}}}_{1}\left(\bar{t}\right)=0.01$. For ${\bar{\dot{w}}}_{1}\left(\bar{t}\right)=0$, due to the dissipation of damping, the vibration amplitude of the mass block gradually decreases and the mass finally rests at the static mode, as shown in Figure 4a,b. For ${\bar{\dot{w}}}_{1}\left(\bar{t}\right)=0.01$, the fiber-mass system also translates with the translating frame, as shown in Figure 4c,d. However, the mass first vibrates and finally stops vibration due to the damping dissipation. It is noted that the equilibrium position of the mass and the length of the LCE fiber vary with time because of the variation of contraction of the LCE fiber translating in the steady linear temperature field. 

### 3.4. Uniformly Accelerated Translational Frame with ${\bar{\dot{w}}}_{1}\left(\bar{t}\right)={\bar{a}}_{m}\bar{t}$

**Figure 5:**
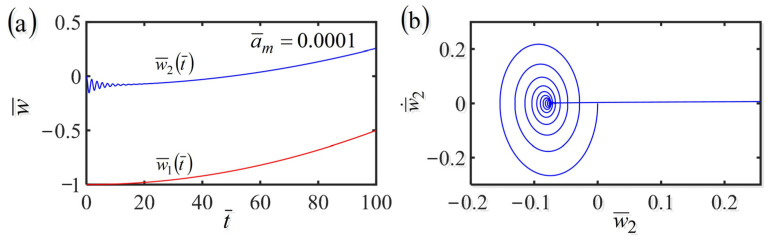
(**a**) Time history and (**b**) phase trajectory of the displacement of the system consisting of fiber and mass in the case of the frame at uniformly accelerated translation in a linear temperature field. The red line represents ${\bar{w}}_{1}\left(\bar{t}\right)$ and the blue line represents ${\bar{w}}_{2}\left(\bar{t}\right)$. Whether the frame is at uniform translation or uniformly accelerated translation, the equilibrium position of the mass and the length of the LCE fiber vary with time because of the variation of contraction of the LCE fiber translating in the linear steady temperature field.

Then, we analyze the dynamics of the fiber-mass system in the case of the frame at uniformly accelerated translation with ${\bar{\dot{w}}}_{1}\left(\bar{t}\right)={\bar{a}}_{m}\bar{t}$. By setting the dimensionless parameters $\bar{K}=10$, $\bar{\alpha }=-0.38$, $\bar{\beta }=1$, ${\bar{a}}_{0}=0.45$, and the accelerated velocity ${\bar{a}}_{m}=0.0001$, Figure 5 plots the time history and phase trajectory of the displacement of the fiber-mass system with uniformly accelerated translational frame in a linear temperature field. A similar conclusion is found in the cases of the frame at uniform translation as that shown in Figure 4c,d. The mass first vibrates and finally stops vibration due to the damping dissipation. Meanwhile, whether the frame is at uniform translation or uniformly accelerated translation, the equilibrium position of the mass and the length of the LCE fiber vary with time because of the variation of contraction of the LCE fiber translating in the steady linear temperature field.

## 4. Dynamics of the Fiber-Mass System with Small Characteristic Time

In this section, we utilize the constitutive model and asymptotic relationship to study the dynamical behaviors of the translating fiber-mass system with small characteristic time $\bar{\tau }<<1$ in the typical cases of the frame at rest, uniform, and accelerating translation. 

### 4.1. Governing Equations

The translating fiber-mass system in a temperature field varying linearly with position with a small characteristic time is represented by Figure 2. It is noted that the system only has two modes for linear damping: converging or diverging [47]. In contrast, the system can continuously vibrate without diverging in practice. [47]. Only the nonlinear damping would be considered in the current study, and it is assumed that:

(25) $$F_{d}\left(\dot{w}\right)=\left(a_{0}+a_{1}\left|\dot{w}\right|\right)\dot{w},$$

where $a_{0}$ and $a_{1}$ are the corresponding damping coefficients. With the definition of the dimensionless parameters ${\bar{F}}_{d}=F_{d}/mg$, ${\bar{a}}_{0}=\frac{a_{0}}{m}\sqrt{\frac{L}{g}}$, and ${\bar{a}}_{1}=\frac{a_{1}L}{m}$, we substitute the Equations (18) and (25) into Equation (19), and the governing equation for the mass block can be rewritten as:

(26) $$\begin{array}{l} {\bar{\ddot{w}}}_{2}\left(t\right)-1+\bar{K}\bar{\alpha }\bar{\beta }\bar{\tau }\frac{1-e^{\bar{\alpha }\bar{\beta }}+\bar{\alpha }\bar{\beta }e^{\bar{\alpha }\bar{\beta }}}{{\left(e^{\bar{\alpha }\bar{\beta }}-1\right)}^{2}} [{\bar{\dot{w}}}_{2}\left(\bar{t}\right)-{\bar{\dot{w}}}_{1}\left(\bar{t}\right)]+\frac{\bar{K}\bar{\alpha }\bar{\beta }}{e^{\bar{\alpha }\bar{\beta }}-1}{\bar{w}}_{2}\left(\bar{t}\right)-\frac{\bar{K}\bar{\alpha }\bar{\beta }e^{\bar{\alpha }\bar{\beta }}}{e^{\bar{\alpha }\bar{\beta }}-1}{\bar{w}}_{1}\left(\bar{t}\right)+\bar{K}\left(\frac{\bar{\alpha }\bar{\beta }}{e^{\bar{\alpha }\bar{\beta }}-1}-1\right)\\ +\left({\bar{a}}_{0}+{\bar{a}}_{1}\left|{\bar{\dot{w}}}_{2}\left(\bar{t}\right)\right|\right){\bar{\dot{w}}}_{2}\left(\bar{t}\right)=0 \end{array}$$

By introducing ${\hat{w}}_{2}\left(\bar{t}\right)={\bar{w}}_{2}\left(\bar{t}\right)+\frac{e^{\bar{\alpha }\bar{\beta }}-1}{\bar{K}\bar{\alpha }\bar{\beta }} [\bar{K}\left(\frac{\bar{\alpha }\bar{\beta }}{e^{\bar{\alpha }\bar{\beta }}-1}-1\right)-1-\bar{K}\bar{\alpha }\bar{\beta }\bar{\tau }\frac{1-e^{\bar{\alpha }\bar{\beta }}+\bar{\alpha }\bar{\beta }e^{\bar{\alpha }\bar{\beta }}}{{\left(e^{\bar{\alpha }\bar{\beta }}-1\right)}^{2}}{\bar{\dot{w}}}_{1}\left(\bar{t}\right)]$, Equation (26) can be rewritten as:

(27) $${\hat{\ddot{w}}}_{2}\left(t\right)+\left({\bar{a}}_{0}+\bar{K}\bar{\alpha }\bar{\beta }\bar{\tau }\frac{1-e^{\bar{\alpha }\bar{\beta }}+\bar{\alpha }\bar{\beta }e^{\bar{\alpha }\bar{\beta }}}{{\left(e^{\bar{\alpha }\bar{\beta }}-1\right)}^{2}}\right){\hat{\dot{w}}}_{2}\left(\bar{t}\right)+{\bar{a}}_{1}\left|{\hat{\dot{w}}}_{2}\left(\bar{t}\right)\right|{\hat{\dot{w}}}_{2}\left(\bar{t}\right)+\frac{\bar{K}\bar{\alpha }\bar{\beta }}{e^{\bar{\alpha }\bar{\beta }}-1}{\hat{w}}_{2}\left(\bar{t}\right)-\frac{\bar{K}\bar{\alpha }\bar{\beta }e^{\bar{\alpha }\bar{\beta }}}{e^{\bar{\alpha }\bar{\beta }}-1}{\bar{w}}_{1}\left(\bar{t}\right)=0.$$

It can be seen from Equation (27) that when the damping coefficient is positive, it dissipates energy during movement. When the damping coefficient is negative, it contributes to the motion of the system because energy input from the environment compensates for the damping dissipation [47,61]. Next, we study the dynamics of the fiber-mass system for three typical cases of the frame at rest, uniform, and accelerating translation through the proposed constitutive model and asymptotic relationship. In order to obtain the exact solution, the constitutive model of Equations (11) and (12) is coupled with the governing Equation (19), and then solved by utilizing the fourth-order Runge–Kutta method. In the calculation of the asymptotic solution, the asymptotic relationship of Equation (27) can be numerically calculated to obtain the relationship between the displacement and time of the mass block and to analyze the translating fiber-mass system.

### 4.2. Static Frame with ${\bar{\dot{w}}}_{1}\left(\bar{t}\right)=0$

**Figure 6:**
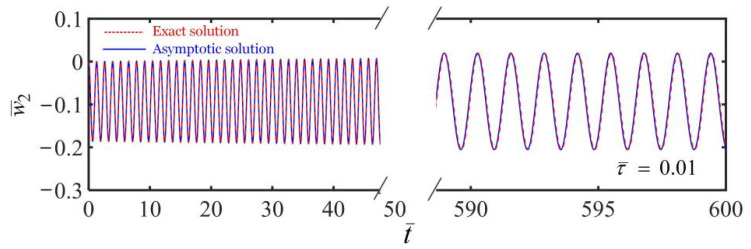
Variation of the displacement of the mass block with time during the self-sustained oscillation in a linear temperature field with a static frame. The LCE fiber in the linear temperature field can stretch or shorten continuously and eventually develop into a periodic self-sustained oscillation.

Figure 6 plots the variation of the displacement of mass with time in the fiber-mass system with a static frame in a linear temperature field. In the numerical calculations, we set the parameters $\bar{K}=20$, $\bar{\tau }=0.01$, $\bar{\alpha }=-0.3$, $\bar{\beta }=1$, ${\bar{a}}_{0}=0.01$, ${\bar{a}}_{1}=0.05$, and the initial state of ${\bar{w}}_{2}\left(0\right)=0$ and ${\bar{\dot{w}}}_{2}\left(0\right)=0$. The blue solid line in Figure 6 shows the asymptotic solution with Equation (27) of the dynamics of the system. Meanwhile, the red dotted line in Figure 6 represents its exact solution of the translating thermally responsive fiber-mass system, which is solved by combining the constitutive model with Equations (11) and (12) and the dynamics governing Equation (19). The comparation between the asymptotic results and the exact numerical results is also presented. It is shown that the two solutions are consistent with each other. As shown in Figure 6, the amplitude of the displacement of the mass block is steady and the mass vibrates periodically in the linear temperature field. This is because during the vibration, the LCE fiber contracts in high temperature and relaxes in low temperature, which leads to continuous stretch or shrink of the LCE fiber and eventually achieves a periodic self-sustained oscillation. During the process of the self-oscillation, the fiber can absorb heat energy from its surrounding environment to sustain its motion due to the compensation for damping dissipation.

**Figure 7:**
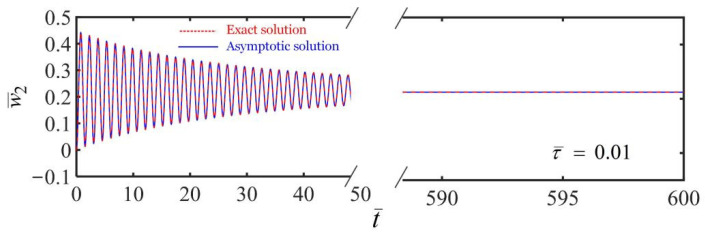
Variation of the displacement of the mass block with time in a linear temperature field with temperature gradient $\bar{\beta }=-1$. It is found that the mass block develops into stationary due to energy dissipation, which is much different from the self-oscillation for the case of $\bar{\beta }=1$.

Here, we take temperature gradient $\bar{\beta }=1$ to describe the linear temperature field distribution, which indicates that the temperature increases from top to bottom. During the movement of the fiber, the LCE fiber contracts in high temperature and relaxes in low temperature, which leads to energy absorption and compensates for damping dissipation. Consequently, the mass block is in the periodic self-excited motion. To further analyze the influence of the temperature gradient, we set the temperature gradient $\bar{\beta }=-1$, which indicates that the temperature decreases from top to bottom. The relationship of the displacement and the time of the mass in the fiber-mass system is shown in Figure 7. It is found that the mass block no longer moves periodically and becomes stationary due to energy dissipation, which is much different from the self-oscillation for the case of $\bar{\beta }=1$. The different behaviors of the dynamic fiber can be understood by the proposed theoretical model. For the case of $\bar{\beta }=-1$, from Equation (27), the damping coefficient is positive, and the fiber engine module represents the damper that dissipates energy during movement and leads to a stationary mass. For the case of $\bar{\beta }=1$, from Equation (27), the damping coefficient is negative, and the fiber engine module can absorb energy from the environment and compensate for the damping dissipation like an engine during the movement, which contributes to the self-oscillation of the system.

### 4.3. Uniformly Translational Frame with ${\bar{\dot{w}}}_{1}\left(\bar{t}\right)=C$

**Figure 8:**
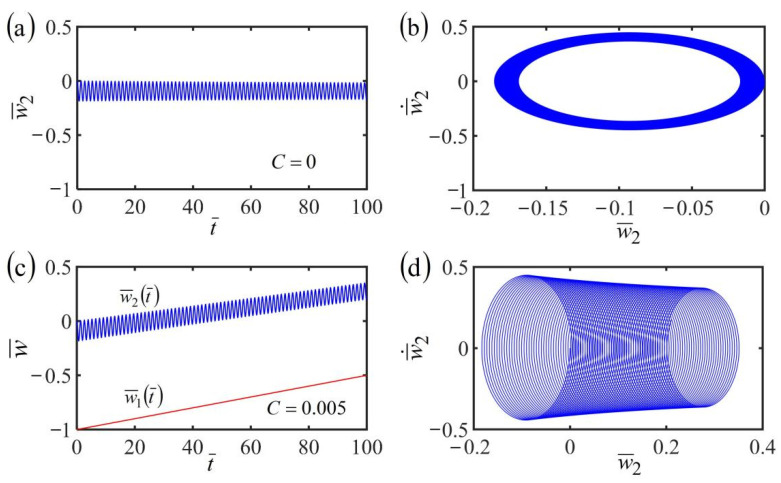
(**a**) Time history of the displacement and (**b**) phase trajectory of the self-oscillation in the case of the frame at rest in a linear temperature field. (**c**) Time history of the displacement and (**d**) phase trajectory of the self-oscillation in the case of the frame at uniform translation in a linear temperature field. The equilibrium position of mass and the length of fiber depend on the translation of the frame.

Next, we consider the dynamics of the translating LCE fiber in the case of a uniform translational frame with ${\bar{\dot{w}}}_{1}\left(\bar{t}\right)=C$. By setting the dimensionless parameters $\bar{K}=20$, $\bar{\tau }=0.01$, $\bar{\alpha }=-0.3$, $\bar{\beta }=1$, ${\bar{a}}_{0}=0.02$, ${\bar{a}}_{1}=0.05$, and the initial state of ${\bar{w}}_{2}\left(0\right)=0$ and ${\bar{\dot{w}}}_{2}\left(0\right)=0$, Figure 8 depicts time histories and phase trajectories of the displacement in the cases of ${\bar{\dot{w}}}_{1}\left(\bar{t}\right)=0$ and ${\bar{\dot{w}}}_{1}\left(\bar{t}\right)=0.005$. Figure 8a,b show that the LCE fiber-mass system first vibrates and eventually develops into periodic self-sustained oscillation for the frame at rest. For the translating frame with ${\bar{\dot{w}}}_{1}\left(\bar{t}\right)=0.005$, the fiber-mass system also translates with the frame, as shown in Figure 8c,d. However, the mass block can also self-oscillate due to the energy compensation between energy input and damping dissipation. Similarly, the equilibrium position of the mass and the length of the LCE fiber also vary with time because of the variation of contraction of the LCE fiber translating in the steady linear temperature field.

### 4.4. Uniformly Accelerated Translational Frame with ${\bar{\dot{w}}}_{1}\left(\bar{t}\right)={\bar{a}}_{m}\bar{t}$

**Figure 9:**
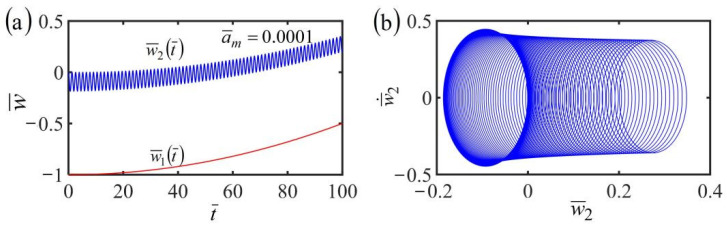
(**a**) Time history of the displacement and (**b**) phase trajectory of the self-oscillation of the fiber-mass system in the case of the frame at uniformly accelerated translation in a linear temperature field. For the case of the frame at uniformly accelerated translation, the mass block can also self-oscillate periodically.

Next, we investigate the case of the translating LCE fiber-mass system in the case of a frame at uniformly accelerated translation with ${\bar{\dot{w}}}_{1}\left(\bar{t}\right)={\bar{a}}_{m}\bar{t}$. By setting the dimensionless parameters $\bar{K}=20$, $\bar{\tau }=0.01$, $\bar{\alpha }=-0.3$, $\bar{\beta }=1$, ${\bar{a}}_{0}=0.02$, ${\bar{a}}_{1}=0.05$, and the accelerated velocity ${\bar{a}}_{m}=0.0001$, the time histories of displacement and phase trajectories of self-oscillation are plotted in Figure 9. For the case of the frame at uniformly accelerated translation, the mass block can also self-oscillate periodically due to the energy compensation between energy input and damping dissipation. Meanwhile, the equilibrium position of the mass and the length of the LCE fiber both vary with time. This is because the LCE fiber translates in the steady linear temperature field and its contraction varies with time. These results are similar to the cases of the frame at uniform translation, as shown in Figure 8c,d. 

## 5. Dynamics of the Fiber-Mass System with Finite Characteristic Time

The asymptotic relationship of Equation (27) and the constitutive model of Equations (11) and (12) have been used to analyze the dynamical behaviors of the translating LCE fiber-mass system with zero $\bar{\tau }$ or small $\bar{\tau }$ in a temperature field varying linearly with position in the above study. In this section, by considering the different finite characteristic times, we study the dynamical behaviors of the translating LCE fiber-mass system in the typical cases of the frame at rest, uniform, and accelerating translation. Since the characteristic time $\bar{\tau }$ is not small, the asymptotic relationship is no longer applicable, and we will only adopt the constitutive model for the numerical calculations. 

**Figure 10:**
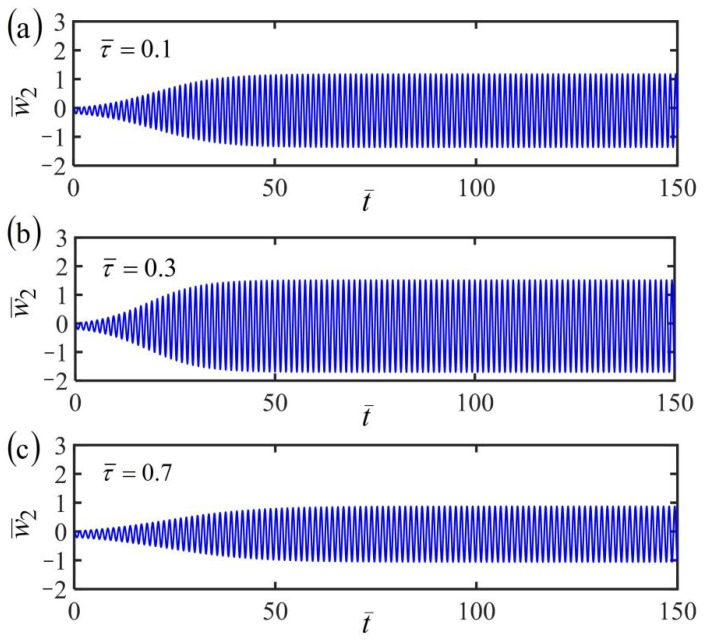
Time histories of the displacement of the fiber-mass system with a static frame in a linear temperature field for (**a**) finite characteristic times $\bar{\tau }=0.1$, (**b**) $\bar{\tau }=0.3$, and (**c**) $\bar{\tau }=0.7$. The amplitude first increases and then decreases obviously with the increasing characteristic time.

**Figure 11:**
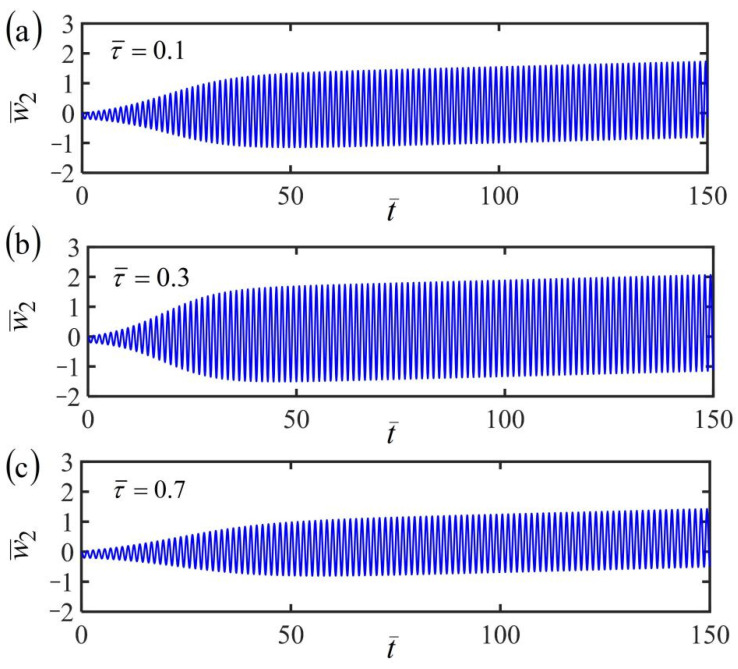
Variations of the displacement of the fiber-mass system with time for the case of a uniform translational frame in a linear temperature field for (**a**) finite characteristic times $\bar{\tau }=0.1$, (**b**) $\bar{\tau }=0.3$, and (**c**) $\bar{\tau }=0.7$. The amplitude first increases and then decreases obviously with the increasing characteristic time.

**Figure 12:**
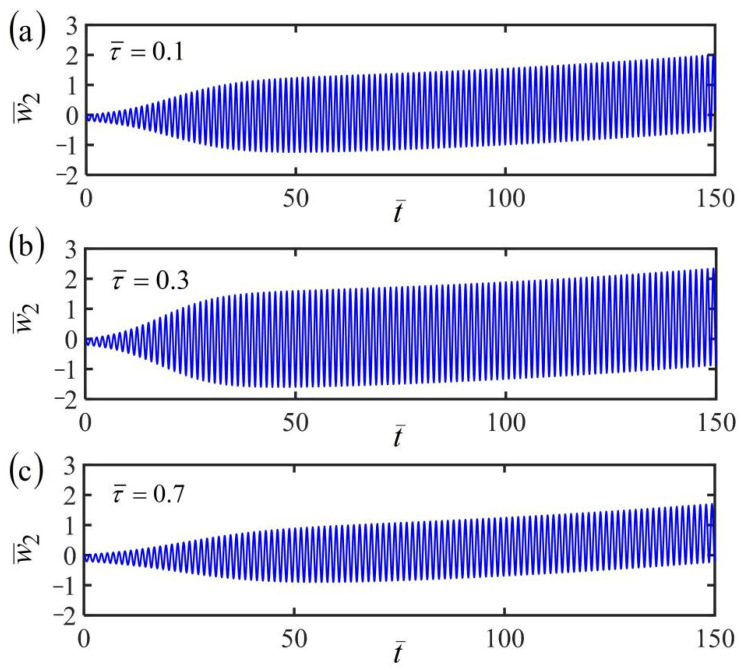
Time histories of the displacement of the fiber-mass system with uniformly accelerated translational frame in a linear temperature field for (**a**) finite characteristic times $\bar{\tau }=0.1$, (**b**) $\bar{\tau }=0.3$, and (**c**) $\bar{\tau }=0.7$. The amplitude first increases and then decreases obviously with the increasing characteristic time.

Figure 10, Figure 11 and Figure 12 plot the time histories of the displacement of mass in the fiber-mass system translating in a linear temperature field for different finite characteristic times $\bar{\tau }$. Three typical cases of the frame at rest, uniform, and accelerating translation are considered in the numerical calculations, by setting the dimensionless parameters $\bar{K}=20$, $\bar{\alpha }=-0.3$, $\bar{\beta }=1$, and ${\bar{a}}_{0}=0.02$, as well as ${\bar{a}}_{1}=0.05$. For the fiber-mass system with a static frame in a linear temperature field, when the characteristic time $\bar{\tau }$ increases, the equilibrium position and frequency are unaffected, while the amplitude first increases and then decreases obviously, as shown in Figure 10. Hence, there exists an optimal characteristic time for the maximum of amplitude, which can provide convenience and guidance for engineering applications. Similarly, the conclusion can also be found in the case of the frame at uniform translation, as shown in Figure 11, and the case of the frame at uniform accelerating translation, as shown in Figure 12. 

For a given finite characteristic time, by comparing Figure 10a, Figure 11a, and Figure 12a of the three cases of the frame at rest, uniform, and accelerating translation, the fiber-mass system remains in the period of self-excited oscillation by the energy input from the ambient linear temperature field to compensate for damping dissipation. Meanwhile, for the frame at uniform translation or uniformly accelerated translation, the equilibrium position of the mass varies with time, which is different from the case of the frame at rest. A similar conclusion is also found in Figure 10b, Figure 11b, and Figure 12b and Figure 10c, Figure 11c, and Figure 12c for different finite characteristic times. These results mean that the finite characteristic time and the frame translation do not affect the self-oscillation. 

## 6. Conclusions

Self-oscillation of a LCE fiber translating in an inhomogeneous temperature field is worth investigating to widen its applications. In this paper, we proposed a theoretic constitutive model and the asymptotic relationship of a LCE fiber translating in a temperature field varying linearly with position and investigated the dynamical behaviors of a fiber-mass system translating in a linear temperature field. The main conclusions are summarized as follows: (i) For zero characteristic time, the LCE fiber-mass system in the linear temperature field vibrates freely and cannot develop into self-oscillation in the three typical cases of the frame at rest, uniform, and accelerating translation. (ii) For a small characteristic time, the LCE fiber-mass system in the linear temperature field can self-oscillate by absorbing energy from the surrounding temperature field to compensate for the damping dissipation. (iii) For a finite characteristic time, the amplitude of the self-oscillation of the LCE fiber-mass system increases first, then decreases as the characteristic time increases, and at an optimal characteristic time, the amplitude can attain the maximum value. (iv) In the three cases of the frame at rest, uniform, and accelerating translation, the equilibrium position of the self-oscillation is different, while its amplitude and frequency are the same. The results are expected to provide some useful recommendations for the design and motion control of micro-robots, motors, and active machines with various structures and applications.

## Figures and Tables

**Table 1:** Material properties and geometric parameters.

Parameter	Definition	Value	Units
$a_{0}$	first damping coefficient	$0\sim 5\times 10^{-5}$	kg/s
$a_{1}$	second damping coefficient	$0\sim 5\times 10^{-5}$	kg/s
$a_{m}$	accelerated velocity of the translational frame	$0\sim 1\times 10^{-4}$	m/s^2^
C	uniform velocity of the translational frame	0~0.01	m/s
g	gravitational acceleration	10	m/s^2^
h	heat exchange coefficient	10~20	W/m^2^/°C
K	spring constant	1~3	N/m
L	original length of fiber	0.1	m
m	mass of the mass block	0.001	kg
R	radius of the thin LCE fiber	$1\times 10^{-5}$	m
α	thermal expansion coefficient	−0.003~−0.005	1/°C
β	temperature gradient	−0.002~0.002	°C/m
$\rho_{c}$	heat capacity of the fiber per unit length	0.01	J/m^2^/°C

**Table 2:** Dimensionless parameters.

**Parameter**	$\bar{K}$	$\bar{\alpha }$	$\bar{\beta }$	${\bar{a}}_{0}$	${\bar{a}}_{1}$	C	${\bar{a}}_{m}$
**Value**	10~30	−0.3~−0.5	−2~2	0~0.5	0~0.5	0~0.01	0~0.001

## Data Availability

The data that support the findings of this study are available upon reasonable request from the authors.

## Nomenclature

$a_{0}$	first damping coefficient of the mass block
$a_{1}$	second damping coefficient of the mass block
$a_{m}$	accelerated velocity of the translational frame
A	initial amplitude of the free vibration of the mass block
C	uniform velocity of the translational frame
$F_{d}\left({\dot{w}}_{2}\right)$	damping force of the mass block
$F_{L}\left(t\right)$	tensional force of the translating LCE fiber
g	gravitational acceleration
h	heat exchange coefficient between the LCE fiber and the environment
K	spring constant of the LCE fiber
L	original length of the LCE fiber
m	mass of the mass block
R	radius of the thin LCE fiber
t	time
$T\left(X,t\right)$	temperature field of the LCE fiber
$T_{\mathrm{ext}}\left(x\right)$	environment temperature field
$T_{L}$	environmental temperature at x=L
$T_{r}$	reference temperature in reference state
$u\left(X,t\right)$	instantaneous displacement of a material point
$w_{1}\left(t\right)$	displacement of the translating frame
${\dot{w}}_{1}\left(t\right)$	velocity of the translating frame
$w_{2}\left(t\right)$	displacement of the free end of the LCE fiber
${\dot{w}}_{2}\left(t\right)$	velocity of the free end of the LCE fiber
X	Lagrangian coordinate of the LCE fiber
x	Eulerian coordinate
$x\left(X,t\right)$	instantaneous position of a material point
α	thermal expansion coefficient of the LCE fiber
β	temperature gradient of the external temperature field
δ	damping coefficient of the free vibration of the mass block
$\varepsilon \left(X,t\right)$	one-dimensional strain of the LCE fiber
$\varepsilon_{T}\left(X,t\right)$	thermally induced strain of the LCE fiber
θ	initial phase angle of the free vibration
$\rho_{c}$	heat capacity of the LCE fiber per unit length
τ	heat transfer characteristic time
$\omega_{0}$	natural angular frequency of the free vibration
